# Oncological Minimally Invasive Surgery

**DOI:** 10.1155/2019/1903297

**Published:** 2019-09-12

**Authors:** Qiang Huo, Qifeng Yang, Reginald M. Gorczynski, Tao Li

**Affiliations:** ^1^Department of Spine Surgery, Zibo Central Hospital Affiliated to Shandong University, Zibo, China; ^2^Department of Breast Surgery, Qilu Hospital of Shandong University, Ji'nan, China; ^3^Transplant Research Division, University Health Network and Toronto General Hospital, Toronto, Canada

Combination of surgical procedure, chemotherapy, radiotherapy, and/or other adjuvant therapies has been widely acknowledged in the treatment of tumors [1]. With the development of medical technologies and equipment, surgical treatment of tumors, especially benign conditions, is much less invasive since it involves much smaller surgical incisions than the corresponding open procedures, which were very popular in the past. Minimally invasive surgery (MIS) originally refers to surgical procedures that limit the size of surgical incisions needed so that the blood loss, wound healing time, associated pain and scarring, hospitalization time, risk of infection, and postsurgical complications are usually much less. Nowadays, many conditions previously requiring open surgery could be treated with minimally invasive procedures. With the help of imaging techniques (such as arthroscopic or laparoscopic techniques) and radiologists, surgeons are able to diagnose, identify internal features, and perform surgical procedures with very small incisions [2].

However, minimally invasive surgical procedures seem not so welcomed or prevalent in malignant medical conditions. Cancerous tissues or cells could spread from an initial or primary site to a different or secondary site, sometimes distantly, within the patient's or host's body. Therefore, surgical treatment puts more emphasis on cleaning up the cancerous tissues rather than minor incisions, and omission of tiny cancerous tissues during surgical removal would lead to fatal results, even for preinvasive lesion or carcinoma in situ sometimes [3].

While, the concept, advantages, and benefits of minimally invasive procedures, which have been mentioned above, should not be ignored in the treatment of cancers. Moreover, a large amount of previous research has indicated that the enlarged extent of operating did not bring increased survival rate. Neoplasms such as early gastric cancer, colon cancer, and esophageal cancer are now preferentially approached with minimally invasive surgery with decreased pain, lower wound infection rates, better postoperative pulmonary function, and shorter recovery time compared with traditional laparotomy [2, 4].

On the other hand, cancer patients at advanced stages are usually poor candidates for more invasive procedures, who may be unable to tolerate open surgery or rounds of external beam radiotherapy. These patients usually suffer a lot during survival, such as intense pain and paraplegia, if no surgical procedure was applicable besides conservative management. To satisfy these patients with relatively mild surgical procedures besides palliative treatment, MIS management is pretty necessary. For example, we have developed a steerable stereotactic injection system of bone cement and corresponding therapeutic strategies which improved survival time and living quality significantly of patients with metastatic epidural spinal cord compression (data not shown).

Although the surgical oncology community was initially slow to adopt these techniques, accumulated sound data showed that minimally invasive techniques could provide equivalent outcomes compared with traditional open approaches in many cases [5]. While controversy still exists regarding the different choices of surgical approaches, minimally invasive or open, for the treatment of cancer [3, 6]. We also welcome harsh criticism from colleagues who preferred traditional surgical approaches to minimally invasive techniques in this special issue. We believed that the introduction of novel surgical tools and digital information technologies will expand the preference for minimally invasive approach to many other cancer operations [7].
